# The Trans-Pacific Partnership: Should We "Fear the Fear"? Comment on "The Trans-Pacific Partnership: Is It Everything We Feared for Health?"

**DOI:** 10.15171/ijhpm.2016.140

**Published:** 2016-10-19

**Authors:** Helen L. Walls, Johanna Hanefeld, Richard D. Smith

**Affiliations:** Faculty of Public Health and Policy, London School of Hygiene and Tropical Medicine, London, UK.

**Keywords:** International Trade, Public Health, Research, Policy, Governance

## Abstract

RLabonté et al entitle their paper in this issue of the *International Journal of Health Policy and Management* "The Trans-Pacific Partnership: Is It Everything We Feared for Health?" Tantalisingly, they do not directly answer the question they pose, and in this commentary, we suggest that it is the wrong question; we should not ‘fear’ the Trans-Pacific Partnership (TPP) at all, rather we should ask how we are to respond. The public health community is right to be concerned with the potential implications of trade and investment agreements (TIAs) for health, particularly with shifts from multilateral to regional/bilateral agreements including provisions with greater risk to public health. But it is critical to understand also the potential health *benefits*, and especially the mitigating policy and governance mechanisms to *respond* to adverse TIA implications. Given entrenched and divergent sectoral worldviews and perspectives between trade and health communities on these issues, achieving the requisite understanding will also likely require characterisation of these perspectives and identification of areas of common understanding and agreed solutions.

Franklin D. Roosevelt famously stated that “the only thing we have to fear is fear itself.” It is tempting to suggest that Labonté et al,^1^ who entitle their paper in this issue of the *International Journal of Health Policy and Management* “The Trans-Pacific Partnership: Is It Everything We Feared for Health?” might reflect on this. The public health community is right to be concerned with the potential implications of trade agreements for health. All too frequently, however, the way that public health researchers engage with work addressing such agreements is taken from a position of hostility, rather than a more objective assessment of the possible opportunities as well as threats that they may represent; and usually uninformed by empirical analysis. Thus, although there is a growing body of literature exploring the possible relationships between trade and investment agreements (TIAs) and health, and suggesting an array of detrimental public health impacts, this work has largely been conceptual in nature. There is an equally strong body of conceptual literature suggesting the positive nature of TIAs for national and global economies, with indirect benefits to health; for example.^2-5^ What is critical is to tackle two key areas. First, the challenge of undertaking high-quality, holistic empirical research in this complex and highly multidisciplinary area to determine the various and net impacts of such agreements stemming from direct and indirect routes over the short-, medium- and long-term. The Labonté et al^1^ article moves us a step closer in this direction. Second, it is critical also to tackle the challenge of developing mechanisms that foster positive impact and govern against negatives.

This need for evidence and mechanisms is becoming more pressing given the rapidly changing nature of the global environment of TIAs, with moves from multilateral to regional and bilateral agreements, including provisions with greater risk to public health. Only a decade ago the focus of the public health community in this area was sharply on the World Trade Organization (WTO), and agreements such as the WTO Agreement on Trade-Related Aspects of Intellectual Property Rights (TRIPS), the WTO General Agreement on Trade in Services (GATS), and the WTO Technical Barriers to Trade (TBT) Agreement.^6^ Yet even then it was clear that greater regional and bilateral development of trading relationships and TIAs was becoming more prominent.^7-9^ Since that time, the impact of the investor-state dispute settlement mechanism (ISDS) has been clearly seen.^10,11^ The concerns with these ‘new generation’ TIAs – notably the recently signed Trans-Pacific Partnership (TPP) between 12 Pacific-Rim countries, and the Transatlantic Trade and Investment Partnership (TTIP) under negotiation between the United States and the European Union (EU) – have been well-documented.^12-16^ But are they to be “feared,” given that fear often paralyses, and a hostile stance may contribute to being isolated from negotiation process and thus, unable to secure beneficial aspects of TIAs?

Labonté et al^1^ do us a great service, as they move beyond the conceptual rhetoric in this area, and provide an analysis of specific treaty text – TPP – for possible health impacts using a prospective health impact analysis (HIA) methodology. This involves the use of a standard protocol for conducting HIAs coupled with a health impact review methodology to create a summary estimation of the most likely significant impacts on health from the cluster of policies making up the TPP. The research reviews the final text of the TPP for potential health implications of intellectual property rights (IPRs), sanitary and phytosanitary provisions (SPS), technical barriers to trade (TBT), ISDS and regulatory coherence provisions on a range of identified issues, and elucidates a number of serious health risks from the TPP. Two of these areas, access to medicines and diet-related health, provide us the opportunity to illustrate some of these points.

As described by Labonté et al,^1^ ‘one of the longest standing public health concerns with post-WTO TIAs has been their potential impact on the price of pharmaceuticals’ (p488-89). A key concern about TIAs has been their potential to extend intellectual property (IP) provisions in a way that limits access to medicines by prolonging or expanding patents and curtailing and limiting competition through cheaper generic medication. A particular focus has been the concern of TIAs extending patent protections for medicines and medical procedures beyond those set out in TRIPS, or by eliminating the flexibilities and safeguards for health provided by TRIPS, to so-called TRIPS-Plus provisions.^17^ Labonté et al point to the TRIPS-Plus provisions in the TTP, as well as to victories in safeguarding TRIPS for health. The TRIPS Agreement and its provisions for public health are essential to safeguarding access to medicines worldwide and the potential impact by TIAs have formed a justified focus for research. At the same time, we know very little indeed about how and why the flexibilities under TRIPS have been implemented or not implemented, particularly by low- and middle-income countries. There is significant scope for empirical research to document and understand the relationship between, and politics of invoking, TRIPS for intellectual property, price and access to medicines.

TIAs are also increasingly linked to changes in diets, nutrition and related health outcomes in countries globally. The recognition of this is also growing. For example, in 2015 the *United Nations’ Standing Committee on Nutrition* published a report on the need for enhancing coherence between trade policy and nutrition action.^18^ Yet little empirical investigation has been undertaken of trade-nutrition relationships, catalysing a review of studies in this area that found relatively few studies, of a generally low standard (H. Walls, S. Cuevas, L. Cornelsen, S. Friel, R. Smith, unpublished data, 2016). This reflects the challenge of conducting quality investigation in this area, and suggests that novel approaches may be useful here. The use of ‘natural experiments’ to investigate these relationships is one such example,^19,20^ although these too are not without significant limitations.

Examination of trade-nutrition relationships requires an understanding of the various pathways of possible impact of TIAs on nutrition, and several frameworks have been proposed (H. Walls, S. Cuevas, L. Cornelsen, S. Friel, R. Smith, unpublished data, 2016).^21-24^ In their paper, Labonté et al identify areas of particular concern for nutrition in the TPP. This includes through the TPP’s TBT and SPS chapters, which: promote harmonisation of technical regulations, standards, and conformity assessments, arguably to the benefit of industry rather than states; increase the scientific evidence required for food safety standards; and create new avenues for vested interests to participate in regulation-setting due to administrative demands. The ISDS provision is also particularly concerning, as the ‘tobacco exceptionalism’ included in the text would not prevent health regulations related to other areas of public health, including those applied to food products, from being challenged by investors. However, it is worth noting that there is a body of literature on the positive impacts of global trade on food security and dietary measures.^25-27^

The challenge of undertaking high-quality trade-health empirical research is vast, but crucial, given the risks of evidence-*un*informed policy. *Ex-ante* studies of potential impact, such as that provided by Labonté et al are an important advance – they may for example help to inform negotiations of possible future problems, and pre-empt major harms to the health of populations. But they remain predictions, and so *ex-post* studies of *actual* impact are also needed to substantiate such prediction. If we are to move beyond the ‘fear’ then this it is critical to understand more fully the impact of potential health risks from TIAs, but also potential health *benefits*. There is also a need for analysis of mitigating policy and governance mechanisms to *respond* to adverse TIA implications.^28^ To achieve this, given entrenched sectoral worldviews and divergent understandings of the issue between, for example, trade and health communities, will also likely require characterisation of these perspectives and identification of areas of common understanding and agreed solutions.^11^ Tantalisingly, Labonté et al do not actually answer the question they pose: “The Trans-Pacific Partnership: Is It Everything We Feared for Health?” We would suggest that it is the wrong question; we should not fear the TPP at all, rather we should ask how we are to respond. And in this, the recommendations and conclusions presented by Labonté et al do help to build a way forward, and we heartily endorse their implementation.

## Ethical issues

Not applicable.

## Competing interests

Authors declare that they have no competing interests.

## Authors’ contributions

RDS conceived of the article. All authors contributed to critical context, manuscript development and writing.
